# Supplementary material for: Two phases of aging separated by the Smurf transition as a public path to death
Source: Sci Rep. 2016 Mar 22;6:23523. doi: 10.1038/srep23523 (PMC4802314; doi:10.1038/srep23523)
Supplement: Supplementary Information [file srep23523-s1.pdf]

Two phases of aging separated by the Smurf transition as a public path to death

E. Dambroise[2,3*] & L. Monnier[4*], L. Ruisheng[4], H. Aguilaniu[4], J.-S. Joly[3], H. Tricoire[1] and M. Rera[1,#]

1. Unité de Biologie Fonctionnelle et Adaptative (BFA) UMR8251 - CNRS - Université Diderot, Sorbonne Paris Cité, 4 Rue Marie Andrée Lagroua Weill Hallé, 75013, Paris, France
2. current address : Equipe "Bases moléculaires et physiopathologiques des ostéochondrodysplasies", U1163 UMR Imagine, Paris, France
3. Equipe CASBAH "Comparative Analysis of Stem cells, Brain Anatomy and Homeostasis", Neuroscience Paris-Saclay Institute (Neuro-PSI), UMR 9197, CNRS, Gif-sur-Yvette, France
4. Ecole Normale Supérieure de Lyon - CNRS - Université de Lyon Claude Bernard - Institut de Génomique Fonctionnelle de Lyon /UMR5262 46, Allée d'Italie, 69364 Lyon, France

*equally contributed to this work

# Correspondence:
Michael Rera, Ph.D.
Phone: +33 1 57 27 79 51
Email: michael.rera@univ-paris-diderot.fr

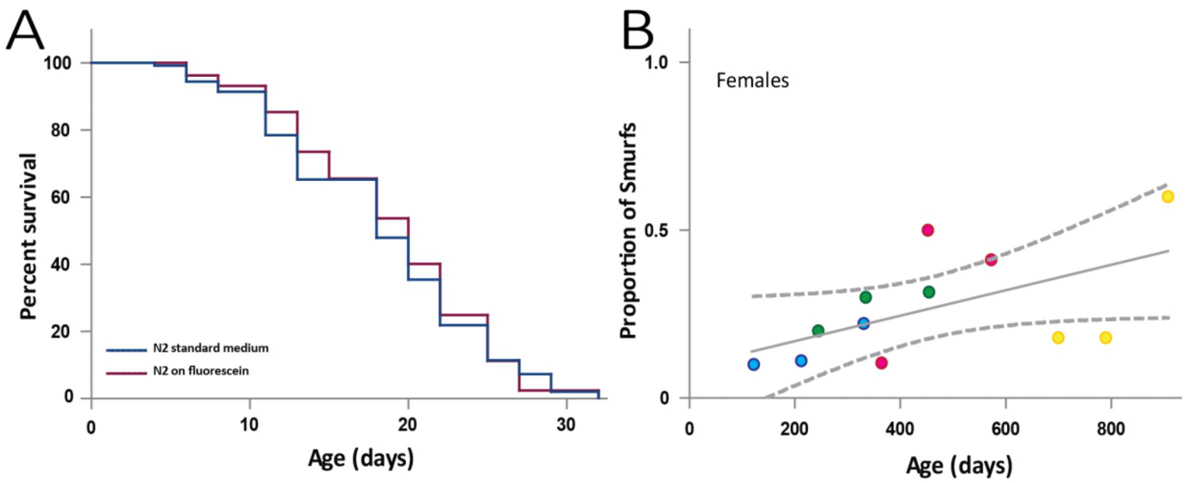

Figure S1: (A) Longevity curves of N2 nematodes maintained either on normal food (T_{50} = 18.2 days, N = 98) or fluorescein containing food (T_{50} = 18.8, N = 126). The curves are not statistically different thus showing that the presence of fluorescein added in the medium does not affect the lifespan of nematodes. (B) Smurf proportion in the 4 different groups of females *D. rerio* used to calculate SIR (0.0003743 ± 0.0001763, R^2 = 0.3107, p = 0.0597) in zebrafish.
